# Ethyl diazoacetate synthesis in flow

**DOI:** 10.3762/bjoc.9.211

**Published:** 2013-09-05

**Authors:** Mariëlle M E Delville, Jan C M van Hest, Floris P J T Rutjes

**Affiliations:** 1 Radboud University Nijmegen, Institute for Molecules and Materials, Heyendaalseweg 135, 6525 AJ Nijmegen, the Netherlands

**Keywords:** diazo compounds, diazotization, ethyl diazoacetate (EDA), flow chemistry, microreactor technology

## Abstract

Ethyl diazoacetate is a versatile compound in organic chemistry and frequently used on lab scale. Its highly explosive nature, however, severely limits its use in industrial processes. The in-line coupling of microreactor synthesis and separation technology enables the synthesis of this compound in an inherently safe manner, thereby making it available on demand in sufficient quantities. Ethyl diazoacetate was prepared in a biphasic mixture comprising an aqueous solution of glycine ethyl ester, sodium nitrite and dichloromethane. Optimization of the reaction was focused on decreasing the residence time with the smallest amount of sodium nitrite possible. With these boundary conditions, a production yield of 20 g EDA day^−1^ was achieved using a microreactor with an internal volume of 100 μL. Straightforward scale-up or scale-out of microreactor technology renders this method viable for industrial application.

## Introduction

Diazo compounds are frequently used versatile building blocks in organic chemistry [1–2]. From this class of compounds diazomethane and ethyl diazoacetate (**1**, EDA) are arguably the synthetically most useful ones. Due to the potentially explosive nature of diazomethane and EDA [3–5], however, synthetic routes that involve large scale batchwise handling of such diazo compounds is generally avoided in industrial processes. With the advent of continuous processing over the past decade, new approaches have appeared to conceptually change the way chemical synthesis is performed. In particular continuous-flow microreactor technology offers multiple advantages over batch chemistry, including the inherently safe conducting of reactions due to the small reactor dimensions, efficient heat transport and excellent control over the reaction conditions [6–8]. While the synthesis of diazomethane has been extensively explored in batch [9] and in continuous-flow reactors [10–11], EDA is synthesized via different routes in batch [12–13], but relatively little is known about continuous-flow approaches [14]. Considering the importance of EDA in a wide variety of reactions e.g. cyclopropanation, X–H insertion, cycloaddition and ylide formation [13,15], and more recently, in the synthesis of valuable compound classes such as β-keto esters [16] and β-hydroxy-α-diazocarbonyl compounds [17], we aimed to develop an inherently safe continuous-flow EDA process using microreactor and separation technology.

**Scheme 1:**
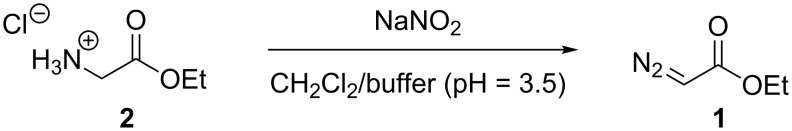
Synthesis of ethyl diazoacetate (**1**).

Ethyl diazoacetate (**1**) can be synthesized in flow via different pathways. Bartrum et al. [18] published a flow synthesis of numerous diazo esters starting from the corresponding arylsulfonylhydrazones, where the diazo moiety was installed through elimination of the sulfone substituent. Additionally, Ley et al. [19] recently prepared a range of α-hydroxy acids in flow starting from the corresponding amino acids, involving diazotization of the amine to the diazonium salt in a biphasic system. Inspired by Ley’s approach, which is significantly more atom efficient than the sulfonylhydrazone pathway, we chose to synthesize EDA (**1**) from glycine ethyl ester (**2**) using readily available sodium nitrite [20] (Scheme 1). Although the diazotization step itself resembles the first step of Ley’s hydroxy acid synthesis, we specifically aimed to produce and isolate the diazo product, which from there can be used for subsequent reactions.

We intended to optimize the process focusing on decreasing the residence time in order to reduce solvent use and gain in throughput. Reaction temperature was considered less of an issue since in an industrial setting energy can generally be efficiently regenerated. In-line phase separation was thought to greatly enhance the usefulness of the EDA flow synthesis. Therefore, the outlet of the microreactor was directly connected to membrane-based phase separator to obtain EDA in the organic phase, which in principle can then be immediately used for either batch [13,15] or continuous-flow [16–17] follow-up reactions. Straightforward scale-up or scale-out of microreactor technology renders this method viable for industrial application.

## Results and Discussion

### Flow synthesis

**Figure 1:**
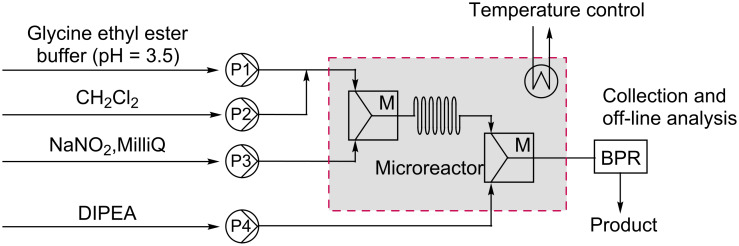
Schematic representation of the microreactor setup.

Ethyl diazoacetate (**1**) was synthesized from glycine ethyl ester (**2**) and sodium nitrite in a biphasic system of dichloromethane and an aqueous sodium acetate buffer. Dichloromethane was chosen as the organic phase to dissolve the water insoluble EDA, because of its low water uptake and low boiling point and its compatibility with potential follow-up reactions. In principle, however, any other organic solvent immiscible with water could be used. The pH of the buffer was set to 3.5 which had been identified by Clark et al. as the optimal pH for the reaction [12]. A schematic representation of the initial microreactor set-up is shown in Figure 1. The box with the dotted line indicates the single-glass microreactor containing two mixing units M of the folding flow type [21]. The reactor temperature was controlled by a Peltier element and sensed by a Pt1000 temperature sensor. At the outlet of the microreactor, a back-pressure regulator (BPR, 40 psi) was attached to guarantee a liquid phase even above boiling temperatures of the solvents. To ensure well-defined reaction times during optimization experiments, neat *N*,*N*-diisopropylethylamine (DIPEA) was added via syringe 4 to efficiently quench the reaction. The collected product (60 μmol) was analyzed by HPLC to establish the conversion of the reaction.

### Univariate optimization

**Figure 2:**
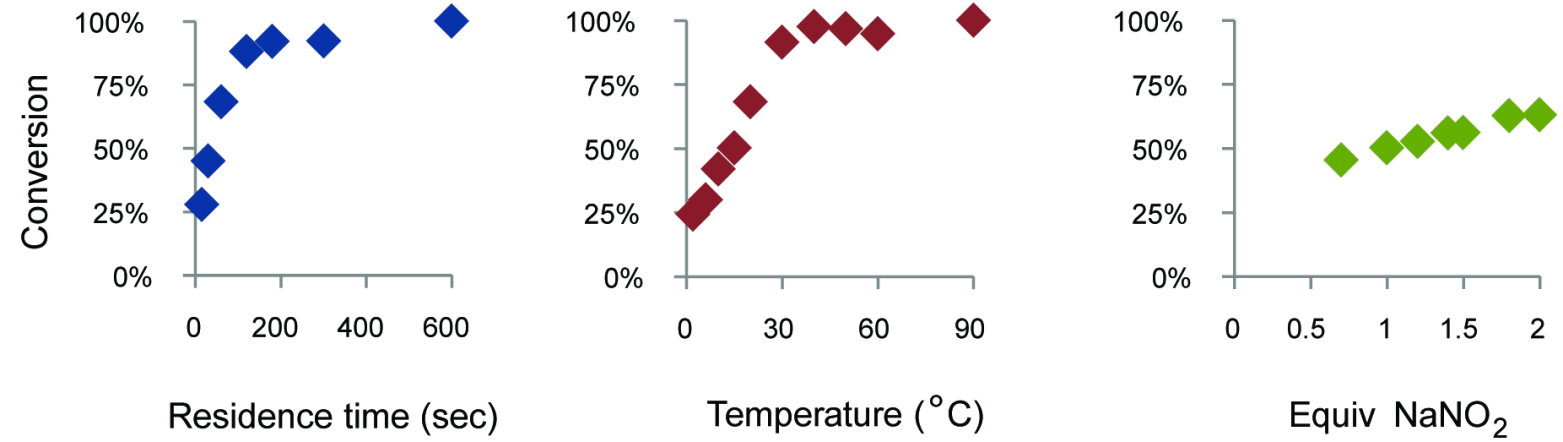
Univariate optimization using 30 s, 15 °C and 1.5 equiv NaNO_2_ as standard.

Determination of the optimal conditions for the reaction started off with investigating the important reaction parameters via a univariate optimization. Based on knowledge obtained from EDA synthesis in batch [12] and other flow reactions [22–23], residence time, temperature and NaNO_2_ stoichiometry were chosen as relevant parameters. Temperature was expected to have a large influence on the rate of the reaction. Shortening the residence time to a minimum would minimize the risk of side reactions and reduce costs, and the reaction should be performed with the smallest amount of NaNO_2_ possible. The results of the univariate optimization are shown in Figure 2. EDA synthesis was shown to be fast, since within 200 seconds complete conversion was obtained at 15 °C. Additionally, the temperature shows a steep increase between 0–30 °C, indicating a large influence of both parameters on the reaction rate. The amount of NaNO_2_ shows only a rather small influence. Based on these univariate optimizations the experimental ranges of the three parameters were determined to investigate the interrelationships via a multivariate optimization.

### Multivariate optimization

**Figure 3:**
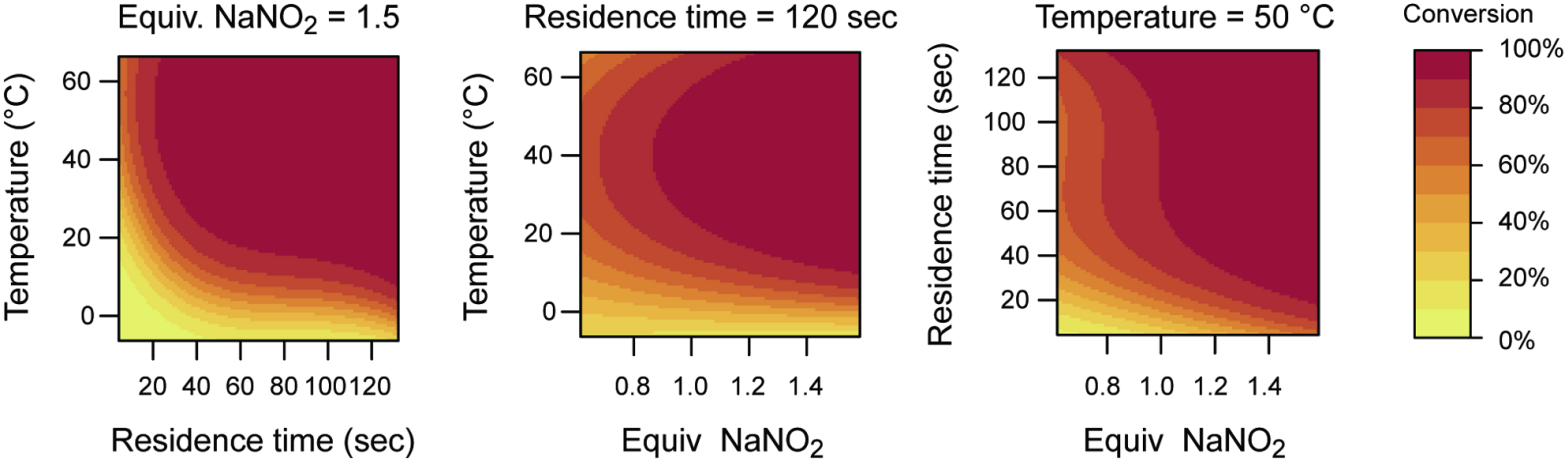
2D-Contour plots of the multivariate optimization.

An experimental design based on a D-optimal algorithm was created from the aforementioned three parameters within their respective ranges, namely 5–120 s, 0–60 °C and 0.7–1.5 equiv of NaNO_2_. Using MATLAB (MathWorks, R2007a), fifty data points were selected of which the corresponding experiments were performed in random order. The resulting HPLC yields were normalized and fitted to a third order polynomial model. In-house-developed FlowFit software [24] was used to calculate the best possible model fit. The results are visualized in 2D-contour plots (Figure 3).

These plots show a rather broad optimum for the conversion of glycine ethyl ester (**2**) into EDA (**1**). The decrease in the upper left corner of the second contour plot can be explained by the high uncertainty of the model at the edge of the plots. As was expected, temperature has a large influence on the reaction rate. The conversion into EDA shows a steep increase with increasing temperature. High temperatures and increasing amounts of NaNO_2_ decrease the residence time to a minimum of 20 seconds while still obtaining complete conversion. Not surprisingly, the minimal amount of NaNO_2_ required is 1 equivalent. We aimed to reach complete conversion into EDA (**1**) maintaining a short residence time with a minimum amount of sodium nitrite, possibly using higher temperatures. Based on these boundary conditions, the optimal parameter settings were fixed at 20 seconds residence time, a temperature of 50 °C using 1.5 equivalents of NaNO_2_. A triple-experiment was performed to prove that this set of optimal parameters indeed provided complete conversion into EDA. The experiment was performed in alternation with two other sets of parameters to rule out potential memory effects. HPLC yields of 95, 96 and 95% for the triple-experiment demonstrate the high reproducibility of the system.

### FLLEX module

**Figure 4:**
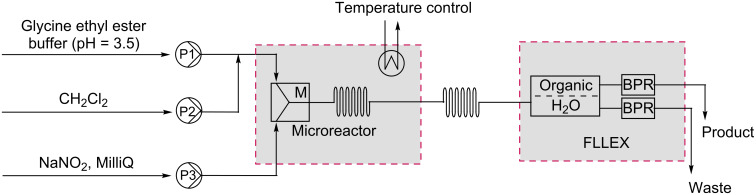
Phase separation using a Flow-Liquid–Liquid-Extraction module (FLLEX) directly coupled to the microreactor.

Having established a microreactor protocol for the continuous-flow synthesis of EDA, the next issue was to separate the product from the biphasic system in which it was collected. In order to increase safety and decrease the hold-up of EDA, the phase separation ideally had to be performed in flow as well. Therefore, a Flow-Liquid–Liquid-Extraction module (FLLEX) [25] was connected to the system [26–27]. The module utilizes a hydrophobic Teflon membrane and two back-pressure regulators (BPRs) to create a pressure difference, which causes the organic layer, in this case dichloromethane, to pass through the membrane resulting in phase separation. A schematic representation of the whole setup is shown in Figure 4.

As the conversion into EDA was quantitative, quenching with DIPEA was no longer required. Between the microreactor and the FLLEX module some additional tubing was used to ensure complete partitioning of the compounds over the two phases. The back pressure of the FLLEX was set to 40 psi, similar to the BPR used previously, and a pressure difference of 0.14 bar. Direct full separation of phases resulted in a clean organic phase containing 409 mg EDA (11 wt % solution in CH_2_Cl_2_, after 30 min of collection) while all salts remain in the aqueous phase. This corresponds roughly to an EDA production of 20 g day^−1^ and a space time yield of 100 kg day^−1^ dm^−3^ as compared to a reported industrial scale batch process yielding EDA in 48 g day^−1^ dm^−3^ [12].

## Conclusion

EDA can be safely synthesized utilizing microreactor and separation technology starting from cheap and readily available starting materials. Optimization of the reaction was aimed at reaching complete conversion into EDA within a minimized residence time using the smallest required amount of sodium nitrite, possibly applying higher temperatures. The optimal reaction conditions identified based on these criteria were a residence time of 20 seconds, a temperature of 50 °C and 1.5 equivalents of NaNO_2_. Repeating the EDA synthesis in flow employing the optimal reaction parameters showed complete conversion and high reproducibility of the results. Additionally, we successfully combined a plug-and-play microreactor setup with a commercially available membrane-based phase separation module to perform a direct in-line extraction of the product. Even in our small set-up (internal volume 100 μL), we were able to generate approximately 20 g of pure EDA per day (11 wt % solution in CH_2_Cl_2_).

## Experimental

### Physical and spectroscopic measurements

NMR spectra were acquired at ambient temperature with a Bruker DMX 300 MHz spectrometer. ^1^H NMR spectra were referenced to TMS or to the residual solvent peak. HPLC analysis was performed using an Agilent 1120 Compact LC, C-18 column, 10% acetonitrile in MilliQ, 254 nm. Pyridine (internal standard) has a retention time of 1.75 min, EDA of 9.67 min.

#### Chip dimensions

Three different microchips were used during the experiments.

1. Single borosilicate glass quench microreactor with an internal volume of 92 μL, a channel width of 600 μm and a channel depth of 500 μm.
2. Single borosilicate glass microreactor with an internal volume of 100 μL, a channel width of 600 μm and a channel depth of 500 μm.
3. Single borosilicate glass quench microreactor with an internal volume of 1 μL, a channel width of 120 μm and a channel depth of 50 μm.

#### Univariate optimization

Solution A: Glycine ethyl ester hydrochloride (40 mmol, 5.6 g) dissolved in 20 mL buffer 1. Solution B: CH_2_Cl_2_. Solution C: NaNO_2_ (60 mmol, 4.1 g) dissolved in 30 mL degassed MilliQ. Solution Q: Neat DIPEA. Buffer 1: Sodium acetate trihydrate (132 mmol, 18.0 g) and pyridine (7.5 mL, internal standard) dissolved in 70 mL MilliQ. Concentrated hydrochloric acid (37%, 12 M) was added until a pH of 3.5 was reached (17 mL), resulting in a buffer with a total volume of 105 mL.

The flow rates and temperatures were set based on predetermined conditions of residence times and temperatures (Table 1). Experiments were performed in a glass microreactor with an internal volume of 92 μL. Solution Q was set at a flow rate 1/3 of the flow rate of solution A. Each experiment had a collection time equal to 30 μL of solution A. The product was collected in 1 mL of acetonitrile and analyzed by HPLC. Results are visualized in Figure 2.

**Table 1:** Conditions of the univariate experiments using 30 s, 15 °C and 1.5 equiv NaNO_2_ as standard.

Time (s)	15	30	60	120	180	300	600	900		
Temperature (°C)	0	5	10	15	20	30	40	50	60	90
Amount of NaNO_2_	0.7	1	1.2	1.4	1.5	1.8	2			

#### Multivariate optimization

Solution A: Glycine ethyl ester hydrochloride (40 mmol, 5.6 g) dissolved in 20 mL buffer 1. Solution B: CH_2_Cl_2_. Solution C: NaNO_2_ (60 mmol, 4.1 g) dissolved in 30 mL degassed MilliQ. Solution Q: Neat DIPEA.

The flow rates and temperatures were set based on predetermined conditions of residence times and temperatures (Table 2). Experiments with a residence time of 5 s were performed in a glass microreactor with an internal volume of 1 μL. For longer residence times, a microreactor with an internal volume of 92 μL was used. Solution Q was set at a flow rate 1/3 of the flow rate of solution A. Each experiment had a collection time equal to 30 μL of solution A. The product was collected in 1 mL of acetonitrile and analyzed by HPLC. Results are visualized in Figure 3 as 2D-contour plots.

**Table 2:** Experiments for the multivariate optimization deduced from a D-optimal experimental design algorithm.

Exp#	Molar ratio	Residence time (s)	Temperature (°C)	Exp#	Molar ratio	Residence time (s)	Temperature (°C)

1	1.5	5	0	26	1.1	120	0
2	1.5	120	60	27	1.5	120	60
3	1.5	45	0	28	0.7	5	0
4	0.7	5	60	29	1.1	15	60
5	1.5	45	60	30	1.1	120	60
6	1.5	120	40	31	1.1	45	60
7	1.5	45	0	32	1.5	5	20
8	0.7	45	0	33	1.5	120	20
9	1.5	15	20	34	0.7	15	0
10	1.1	5	20	35	0.7	5	20
11	0.7	45	20	36	1.1	5	60
12	1.5	120	0	37	1.5	15	60
13	1.1	15	0	38	0.7	45	60
14	0.7	5	60	39	1.1	120	60
15	0.7	120	40	40	1.1	120	40
16	0.7	45	60	41	1.1	5	40
17	1.5	120	0	42	0.7	5	40
18	1.5	5	60	43	1.1	5	0
19	1.5	45	40	44	0.7	120	60
20	0.7	120	60	45	1.1	120	0
21	1.5	5	0	46	0.7	15	40
22	1.5	5	60	47	0.7	5	0
23	1.1	45	0	48	0.7	120	0
24	1.1	120	20	49	0.7	120	20
25	1.5	5	40	50	0.7	120	0

#### FLLEX experiment

Solution A: Glycine ethyl ester hydrochloride (10 mmol, 1.4 g) dissolved in 5 mL buffer 2. Solution B: CH_2_Cl_2_. Solution C: NaNO_2_ (15 mmol, 1.0 g) dissolved in 5 mL degassed MilliQ. Buffer 2: Sodium acetate trihydrate (100 mmol 13.6 g) dissolved in 80 mL MilliQ. Concentrated hydrochloric acid (37%, 12 M) was added until a pH of 3.5 was reached (7 mL). Additional MilliQ was added to obtain a total volume of 100 mL of buffer.

Solution A (86.25 μL/min) was combined in a stainless steel T-splitter with solution B (172.5 μL/min). The biphasic mixture immediately entered the glass microreactor (internal volume: 100 µL) where it was mixed with solution C (86.25 μL/min). The reaction was performed at 50 °C. After the reaction, the mixture was passed through 15 μL of FEP-tubing (ID = 254 μm) before entering the FLLEX module where phases were separated (40 psi, Δ*p* = 0.14 bar). The set-up was stabilized for 2 min before collecting for 30 min. EDA was obtained as a solution in CH_2_Cl_2_ (1.52 g). According to ^1^H NMR analysis, clean EDA was obtained. Based on the residual solvent peak in the ^1^H NMR spectrum it was calculated to be a 27 wt % solution of EDA in CH_2_Cl_2_ meaning 409 mg of pure EDA.
